# Short-coupled variant of torsade de pointes: A systematic review of case reports and case series

**DOI:** 10.3389/fcvm.2022.922525

**Published:** 2022-08-12

**Authors:** Guangqiang Wang, Lin Zhong, Hongxia Chu, Chunxiao Wang, Xuefeng Zhu

**Affiliations:** Department of Cardiology, The Affiliated Yantai Yuhuangding Hospital of Qingdao University, Yantai, China

**Keywords:** short-coupled variant of torsade de pointes, first-coupling interval, QRS duration of ventricular extrasystole, implantable cardioverter defibrillator, systematic review

## Abstract

**Background:**

The short-coupled variant of torsade de pointes (scTdP) is characterized by a particular electrocardiogram (ECG) pattern that shows a short-coupling interval of the initial Tdp beat and that can degenerate into ventricular fibrillation without the presence of structural heart disease. However, its etiology, epidemiology, clinical characteristics, underlying mechanism, treatment, and prognosis remain unclear. This study aimed to systematically review case reports and series of scTdP to synthesize existing data on the demography, clinical characteristics, ECG features, management, and outcomes.

**Methods:**

A literature search was conducted for eligible published articles using the Medline, Embase, and PubMed databases. All eligible case reports and case series were included without any language restrictions. SPSS 24 was used for statistical analysis.

**Results:**

A total of 22 case reports and 103 case series of patients with scTdP were identified and included in the analysis. All selected cases had acceptable quality of evidence. Most young patients without sex differences had no trigger or a negative programmed simulation. The ECGs of all selected patients showed a short first-coupling interval (302 ± 62 ms) and a long QRS duration of ventricular extrasystole (VE) (135 ± 17 ms). The first coupling interval levels and QRS duration levels of VE were significantly longer and wider in patients with scTdP originating from the right ventricular outflow tract (RVOT) than in those with scTdP originating from the Purkinje fibers (380 ± 70 vs. 274 ± 28 ms, *P* < 0.001; 147 ± 8 vs. 131 ± 17 ms, *P* < 0.001), respectively. The receiver operating characteristic curve showed that the optimal cutoff values of the first coupling interval triggering TdP and QRS duration of VE were more than 319 ms and 141 ms (92% sensitivity, 95.7% specificity; 82.6% sensitivity, 77.8% specificity) for predicting the RVOT origin, respectively. The Kaplan-Meier survival curve revealed increased survival in patients with implantable cardioverter defibrillator (ICD) implantation than in patients without ICD implantation (log-rank =10.127, *P* = 0.001).

**Conclusion:**

Some agreements were confirmed in selected case reports regarding the clinical features, diagnosis, and management of scTdPs. Further large-scale and long-term follow-up studies are required to clarify the existing arrhythmogenic entities.

## Introduction

Idiopathic polymorphic ventricular tachycardia (PMVT)/ventricular fibrillation (VF) is the leading cause of unexplained sudden cardiac death (SCD) in the absence of structural heart disease, particularly in young adults (1). The short-coupled variant of torsades de pointes (scTdP) is a rare cause of idiopathic PMVT/VF and is defined as a new electrocardiogram (ECG) entity that exhibits TdP/VF secondary to a short-coupled premature ventricular complex (PVC) with a normal QT interval, mimicking the R-on-T phenomenon (2). TdP, which means twisting of the points, is a potentially life-threatening form of PMVT, which appears on the ECG as a characteristic beat-to-beat varying QRS morphology that is prone to spontaneous reversal. Occasionally, the clinical presentation of TdP is an electrical storm, that is, a cluster of arrhythmic episodes that sometimes degenerates into VF (3). TdP is usually not induced by programmed electrical stimulation during electrophysiological studies. In 1994, Leenhardt et al. first described a series of 14 patients with normal heart structure and a history of syncope, whose electrocardiographic monitoring showed TdP with normal QT intervals initiated by ventricular extrasystole (VE) with a short coupling interval (200–300 ms) (2). Despite the unique ECG features at the TdP onset, other ECG findings specific to Brugada, long QT, or short QT syndrome are lacking. Therefore, it is often difficult to diagnose scTdP after the disappearance of PVCs. ScTdP should be considered as a diagnosis when the etiology of aborted SCD is unknown. Thus, the clinical features of this disease differ from those of long or short QT syndrome in many respects, and the underlying mechanisms have not yet been fully elucidated. In recent years, few reports have been published on this disease. It is important to identify this characteristic electrocardiographic pattern to prevent SCD. In the long term, the spontaneous behavior of arrhythmia is unpredictable. Placement of an automatic implantable cardioverter defibrillator (ICD) is the only confirmed therapy, since no medication can entirely prevent SCD in this disease. Verapamil is the only effective drug that can partially suppress arrhythmias, but it does not prevent SCD. If ventricular arrhythmia recurs despite drug therapy, catheter ablation to initiate premature ventricular beats may be warranted. The feasibility of ablation has been demonstrated in a small series of patients in expert centers, and long-term follow-up data on catheter ablation are lacking. Successful ablation does not invalidate the need for an ICD.

As this is an uncommon condition, there are only short descriptive series and isolated case reports. Our aim was to systematically review case reports and series of scTdP to synthesize existing data on the demography, clinical characteristics, ECG features, management, and outcomes about the disease.

## Methods

This systematic review was conducted according to the Preferred Reporting Items for Systematic Reviews and Meta-analyses (PRISMA) guidelines.

### Search strategy

**Figure 1:**
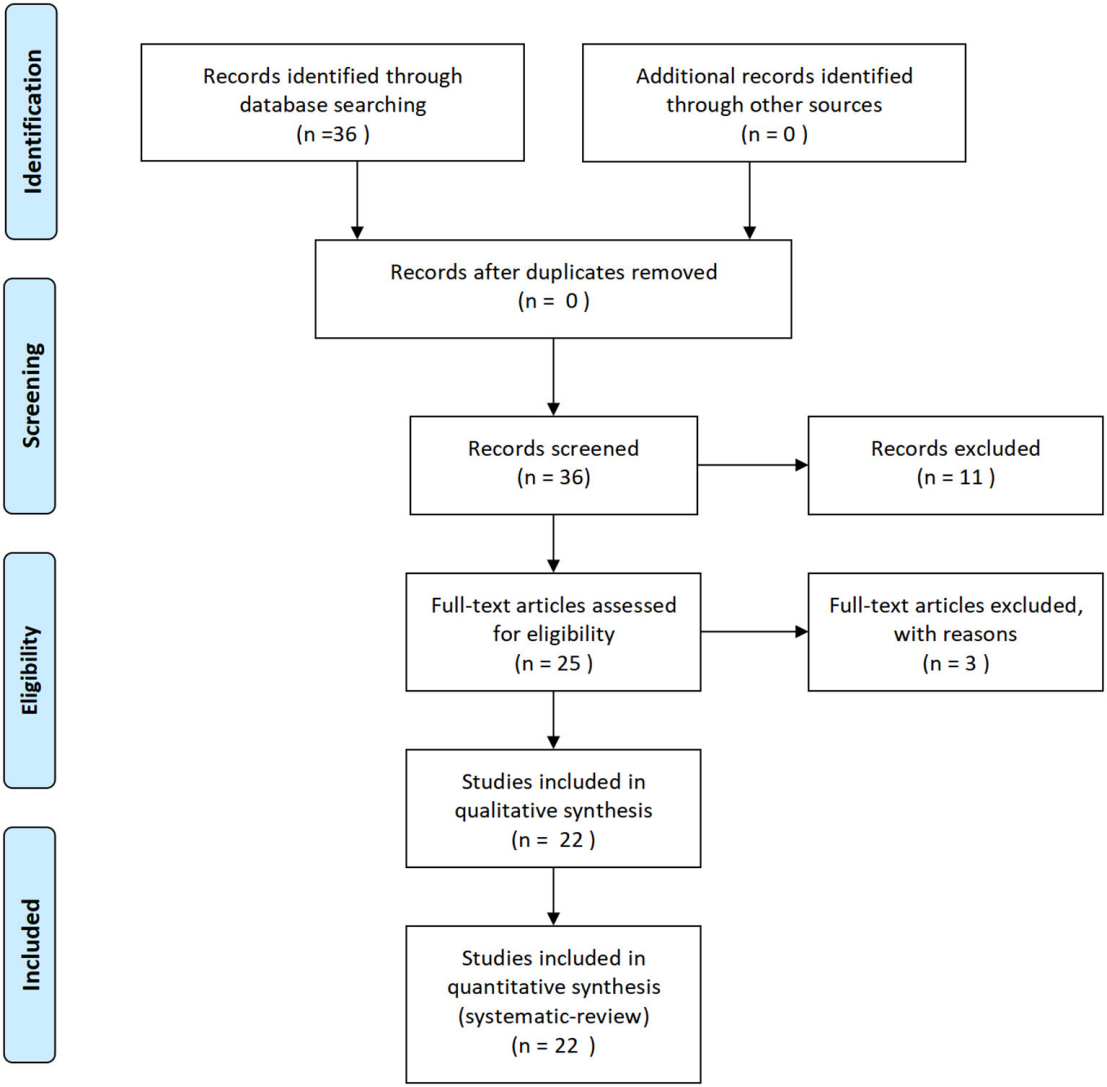
Flow diagram.

A literature search was performed for eligible articles published between January 1994 and December 2021 using the MEDLINE/PubMed and Embase databases. Subsequently, we performed a search using the term “short-coupled variant of torsade de pointes.” The search strategy yielded a total of 36 articles. Eligibility of the case reports was determined by assessing the titles and abstracts. In order to find additional qualifying reports, the reference lists of the included studies and related literature were manually checked. The detailed PRISMA flow diagram is shown in Figure 1.

### Eligibility criteria

All eligible case reports and case series from around the world were included, without any language restrictions. For this review, the inclusion criteria were (1) age and sex of the scTdp patients, (2) clinical and ECG features, (3) results of electrophysiological studies, (4) specific treatment strategies, and (5) patient outcomes. Articles, such as review articles, hypothesis articles, and commentaries, were discarded.

### Study selection and quality assessment

The titles and abstracts of studies from the aforementioned databases were evaluated by three authors (GQW, CXW, and XFZ). The authors assessed the studies based on predetermined eligibility criteria. The critical appraisal checklist for case reports developed by Moola et al. (4) was used to perform a quality check of the systematic review of case reports. If five of the eight evaluation criteria were met, the quality was judged to be sufficient. All researchers agreed on the included studies.

### Data extraction

From these selected studies, three authors (GQW, HXC, and CXW) manually retrieved the data. The following details were extracted from each report: author, country of origin, study design, sample size, mean age, sex, past medical history, family history of sudden death, presenting symptoms, ECG findings, electrophysiological study, treatment, recurrence, follow-up, and outcomes.

### Statistical analysis

Continuous variables are presented as mean ± standard deviation, and categorical variables are expressed as frequency and percentage. A chi-square test and Fisher's exact probability test were used to compare qualitative parameters, and Student's *t*-test and the Mann-Whitney *U*-test were used to compare quantitative parameters. A receiver operating characteristic (ROC) curve was used to evaluate the sensitivity and specificity of the scTdP test. An area under the ROC curve (AUC) of 1.0 indicates perfect discrimination, whereas an area of 0.5 indicates that the test discriminates no better than chance. Kaplan-Meier analysis was performed to plot survival curves. The log-rank test was used to compare event-free survival between groups. In all statistical tests, *P* < 0.05 was regarded as statistically significant. All statistical analyses were performed using SPSS software version 24.0.

## Results

### Study selection

In this review, 103 patients diagnosed with scTdP were included from 22 published studies (2, 3, 5–24). The median age of the included patients was 38 ± 12 years, and 52% of them were female. Most of the reports were from Asia (54.5%), followed by Europe (41%) and America (4.5%). Table 1 presents the patients demographic and clinical characteristic features of each report.

**Table 1:** Characteristics of the case reports.

**References**	**Country**	**Study design**	**Patient (*n*)**	**Age (y, mean ±SD)**	**Female (*n*, %)**	**High risk factors (*n*, %)**	**Structural heart disease (*n*, %)**	**Family history of cardiac disease or sudden death (*n*, %)**	**Emotion and exercise (*n*, %)**	**Syncope (*n*, %)**	**Sudden cardiac arrest (*n*, %)**	**Exercise testing (-) (*n*, %)**	**Provocative testing (-) (*n*, %)**	**Genetic testing**	**QT interval**	**First coupling interval (ms, mean ±SD)**	**Programmed stimulation (+) (*n*, %)**	**QRS duration of ventricular extrasystole (ms, mean ± SD)**	**ICD (*n*, %)**	**Arrhythmia recurrence (*n*, %)**	**Follow-up (months, mean ±SD)**	**Alive (*n*, %)**
Leenhardt (2)	France	Case report	21	33 ± 11	10 (48)	1 (5)	0 (0)	5 (24)	3 (18)	20 (95)	1 (5)	9 (64)	12 (86)	NA	Normal	297 ± 41	2 (14)	NA	4 (19)	9 (43)	52 ± 51	15 (71)
Ruan and Wang (9)	China	Case report	3	36 ± 5	2 (67)	0 (0)	0 (0)	0 (0)	0 (0)	3 (100)	0 (0)	NA	2 (67)	NA	Normal	377 ± 25	NA	NA	0 (0)	1 (33)	7 ± 6	2 (67)
Shiga et al. (11)	Japan	Case report	1	41	0 (0)	0 (0)	0 (0)	0 (0)	0 (0)	1 (100)	0 (0)	NA	0 (0)	NA	Normal	240	1 (100)	156	1 (100)	1 (100)	60	1 (100)
Haïssaguerre et al. (10)	France, Japan, Czech Republic, UK and Brazil	Case report	27	41 ± 14	14 (52)	0 (0)	0 (0)	6 (22)	0 (0)	17 (63)	0 (0)	27 (100)	27 (100)	NA	Normal	297 ± 41	10 (37)	129 ± 18	23 (85)	3 (11)	24 ± 28	27 (100)
Takeuchi et al. (14)	Japan	Case report	1	51	0 (0)	0 (0)	0 (0)	0 (0)	0 (0)	1 (100)	0 (0)	NA	1 (100)	NA	Normal	280	0 (0)	120	1 (100)	0 (0)	6	1 (100)
Noda et al. (8)	Japan	Case control study	16	39 ± 10	9 (56)	0 (0)	0 (0)	1 (6)	0 (0)	11 (69)	0 (0)	16 (100)	16 (100)	NA	Normal	403 ± 21	3 (19)	148 ± 8	1 (6)	0 (0)	54 ± 39	16 (100)
Viskin et al. (19)	Israel	Case report	3	48 ± 11	3 (100)	0 (0)	0 (0)	0 (0)	0 (0)	1 (33)	0 (0)	3 (100)	3 (100)	NA	Normal	350 ± 20	0 (0)	149 ± 7	2 (67)	0 (0)	42 ± 48	3 (100)
Yamazaki et al. (20)	Japan	Case report	1	21	0 (0)	0 (0)	0 (0)	0 (0)	0 (0)	0 (0)	0 (0)	1 (100)	1 (100)	NA	Normal	300	0 (0)	120	0 (0)	0 (0)	36	1 (100)
Bogaard et al. (16)	Netherlands	Case report	1	36	0 (0)	0 (0)	0 (0)	0 (0)	0 (0)	1 (100)	0 (0)	NA	1 (100)	NA	Normal	240	0 (0)	120	1 (100)	0 (0)	6	1 (100)
Chiladakis et al. (3)	Greece	Case report	1	50	1 (100)	0 (0)	0 (0)	0 (0)	0 (0)	1 (100)	0 (0)	NA	1 (100)	NA	Normal	290	0 (0)	120	1 (100)	0 (0)	3	1 (100)
Van den branden et al. (12)	Netherlands	Case report	1	51	0 (0)	1 (100)	0 (0)	0 (0)	0 (0)	0 (0)	1 (100)	1 (100)	NA	NA	Normal	240	NA	160	1 (100)	0 (0)	12	1 (100)
Chokr et al. (6)	Brazil	Case report	4	32 ± 16	4 (100)	0 (0)	0 (0)	0 (0)	3 (75)	3 (75)	0 (0)	2 (50)	2 (50)	NA	Normal	300 ± 43	0 (0)	155 ± 13	3 (75)	1 (25)	71 ± 90	4 (100)
Hayama et al. (15)	Japan	Case report	1	38	0 (0)	0 (0)	0 (0)	0 (0)	0 (0)	1 (100)	0 (0)	NA	1 (100)	(-)	Normal	280	0 (0)	142	1 (100)	0 (0)	15	1 (100)
Jastrzebski et al. (5)	Kraków	Case report	5	43 ± 19	4 (80)	1 (20)	1 (20)	0 (0)	0 (0)	5 (100)	0 (0)	1 (20)	NA	NA	Normal	303 ± 38	NA	130 ± 17	5 (100)	3 (60)	51 ± 29	5 (100)
Kondo et al. (23)	Japan	Case report	1	19	0 (0)	0 (0)	0 (0)	0 (0)	0 (0)	1 (100)	0 (0)	NA	1 (100)	(-)	Normal	300	0 (0)	128	1 (100)	0 (0)	8	1 (100)
Godinho et al. (13)	Portugal	Case report	1	49	1 (100)	0 (0)	0 (0)	0 (0)	0 (0)	1 (100)	0 (0)	NA	1 (100)	NA	Normal	280	0 (0)	160	1 (100)	0 (0)	6	1 (100)
Fujii et al. (17)	Japan	Case report	6	38 ± 9	3 (50)	0 (0)	0 (0)	0 (0)	0 (0)	6 (100)	0 (0)	1 (17)	3 (50)	(+)	Normal	426 ± 21	3 (50)	138 ± 16	6 (100)	3 (50)	62 ± 25	6 (100)
Kimura et al. (18)	Japan	Case report	1	40	1 (100)	0 (0)	0 (0)	1 (100)	0 (0)	1 (100)	0 (0)	0 (0)	1 (100)	(+)	Normal	280	0 (0)	110	1 (100)	0 (0)	NA	1 (100)
Kajiyama et al. (24)	Japan	Case report	1	40	0 (0)	0 (0)	0 (0)	0 (0)	0 (0)	1 (100)	0 (0)	NA	1 (100)	(+)	Normal	250	0 (0)	130	1 (100)	1 (100)	24	1 (100)
Sonoda et al. (22)	Japan	Case report	1	38	0 (0)	0 (0)	0 (0)	0 (0)	0 (0)	1 (100)	0 (0)	NA	1 (100)	(+)	Normal	280	0 (0)	120	1 (100)	0 (0)	NA	1 (100)
Steinfurt et al. (21)	Germany, USA and Netherlands	Case report	5	37 ± 13	2 (40)	0 (0)	0 (0)	1 (20)	0 (0)	1 (20)	4 (80)	NA	5 (100)	NA	Normal	262 ± 20	0 (0)	126 ± 5	5 (100)	0 (0)	32 ± 37	5 (100)
Touat-Hamici et al. (7)	France	Case report	1	35	0 (0)	0 (0)	0 (0)	1 (100)	0 (0)	1 (100)	0 (0)	1 (100)	NA	(+)	Normal	280	NA	120	0 (0)	1 (100)	72	0 (0)
Total	-	-	103	38 ± 12	54 (52)	3 (3)	1 (1)	15 (15)	6 (6)	78 (76)	6 (6)	62 (78)	80 (91)	–	Normal	302 ± 62	19 (22)	135 ± 17	60 (58)	23 (22)	40 ± 41	95 (92)

ICD, Implantable cardioverter defibrillator; SD, Standard deviation; NA, Not applicable.

### Evaluating the risks of biases

Table 2 shows the risk of bias evaluated in this study by using the critical appraisal checklist for case reports. In the evaluated case reports, the demographic characteristics of selected patients, medical history, current clinical status, diagnostic test or evaluation method, and results were all appropriately reported.

**Table 2:** Critical appraisal checklist for case reports included in this review.

**Critical appraisal checklist**	**Leenhardt et al. (2)**	**Ruan and Wang (9)**	**Shiga et al. (11)**	**Haïssaguerre et al. (10)**	**Takeuchi et al. (14)**	**Noda et al. (8)**	**Viskin et al. (19)**	**Yamazaki et al. (20)**	**Bogaard et al. (16)**	**Chiladakis et al. (3)**	**Van den branden et al. (12)**	**Chokr et al. (6)**	**Hayama et al. (15)**	**Jastrzebski et al. (5)**	**Kondo et al. (23)**	**Godinho et al. (13)**	**Fujii et al. (17)**	**Kimura et al. (18)**	**Kajiyama et al. (24)**	**Sonoda et al. (22)**	**Steinfurt et al. (21)**	**Touat-Hamici et al. (7)**
1. Were patient's demographic characteristics clearly described?	Yes	Yes	Yes	Yes	Yes	Yes	Yes	Yes	Yes	Yes	Yes	Yes	Yes	Yes	Yes	Yes	Yes	Yes	Yes	Yes	Yes	Yes
2. Was the patient's history clearly described and presented as a timeline?	No	Yes	Yes	No	Yes	Yes	Yes	Yes	Yes	Yes	Yes	Yes	Yes	Yes	Yes	Yes	Yes	Yes	Yes	Yes	Yes	Yes
3. Was the current clinical condition of the patient on presentation clearly described?	No	No	Yes	No	Yes	No	No	No	Yes	Yes	No	No	Yes	No	Yes	Yes	No	Yes	Yes	Yes	No	No
4. Were diagnostic tests or assessment methods and the results clearly described?	Yes	Yes	Yes	Yes	Yes	Yes	Yes	Yes	Yes	Yes	Yes	Yes	Yes	Yes	Yes	Yes	Yes	Yes	Yes	Yes	Yes	Yes
5. Was the intervention(s) or treatment procedure(s) clearly described?	Yes	Yes	Yes	Yes	Yes	Yes	Yes	Yes	Yes	Yes	Yes	Yes	Yes	Yes	Yes	Yes	Yes	Yes	Yes	Yes	Yes	Yes
6. Was the post-intervention clinical condition clearly described?	Yes	Yes	Yes	Yes	Yes	Yes	Yes	Yes	Yes	Yes	Yes	Yes	Yes	Yes	Yes	Yes	Yes	Yes	Yes	Yes	Yes	Yes
7. Were adverse events (harms) or unanticipated events identified and described?	Yes	Yes	Yes	Yes	Yes	Yes	Yes	Yes	Yes	Yes	Yes	Yes	Yes	Yes	Yes	Yes	Yes	Yes	Yes	Yes	Yes	Yes
8. Does the case report provide take away lessions?	Yes	Yes	Yes	Yes	Yes	Yes	Yes	Yes	Yes	Yes	Yes	Yes	Yes	Yes	Yes	Yes	Yes	Yes	Yes	Yes	Yes	Yes
Overall appraisal: include, exclude, and seek further information	Include	Include	Include	Include	Include	Include	Include	Include	Include	Include	Include	Include	Include	Include	Include	Include	Include	Include	Include	Include	Include	Include

### Clinical characteristics

Syncope was the most prevalent presenting symptom in 76% of patients with scTdP, followed by sudden cardiac arrest (6%). A majority of patients without sex differences had no high-risk factors for coronary artery disease (97%), no presence of structural heart disease (99%), no family history of cardiac disease or sudden death (85%), and no emotional stress (94%) (Table 1). The exercise stress test and provocative testing were negative in 78 and 91% of the enrolled patients, respectively. In addition, the programmed simulation was also negative in 78% of the selected patients.

### ECG findings

**Figure 2:**
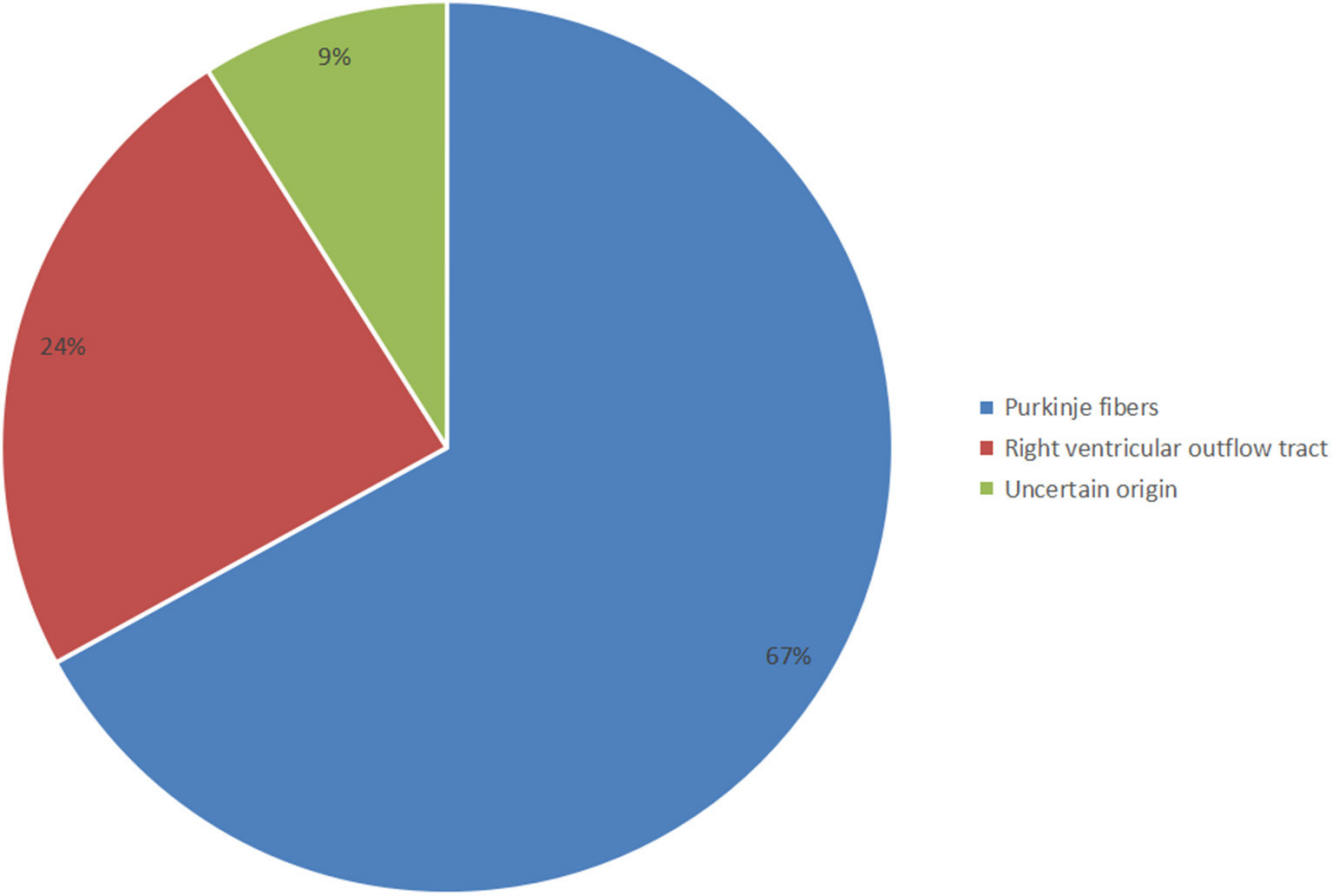
Distribution of the different origins of short-coupled variant of torsade de pointes (scTdP).

Significant clues suggesting the PVC triggering TdP were often found upon analysis of telemetry and ambulatory monitor tracings. Then, a meticulous inspection of the 12-lead ECG indicating different characteristic morphologies of PVCs should be done to distinguish the PVC origin between the right ventricular outflow tract (RVOT) and Purkinje fibers. The 12-lead ECGs of all selected patients showed sinus rhythm, normal QRS-ST-T morphology, and QT intervals. Among them, the inferior J-wave in only two cases may not be a critical finding but rather a sign of clinical or genetic heterogeneity (17). The first coupling interval was 302 ± 62 ms (<400 ms) and the QRS duration of VE was 135 ± 17 ms (<153 ms). Around 24% of selected patients showed a left bundle branch block (LBBB) pattern with a right axis deviation, suggesting that the origin of the PVCs was RVOT localizing along the RV papillary muscle, carrying within its muscular bundle a major fascicle of the right bundle branch. Most of the selected patients (67%) showed a right bundle branch block (RBBB) or LBBB configuration with a left axis deviation, suggesting that the origin of the PVCs was the Purkinje fibers (Figure 2). However, there were only a few cases in which the PVC origin estimated by the ECG pattern differed from the site of successful PVC ablation (10, 21).

### PVC origins

The PVC origins could not be identified because of the fragmentary inspection of recorded ECGs in nine patients. In total, the data of 94 patients with different origins of Purkinje fibers and RVOT were analyzed (Table 3). Significant differences were found in the values of the selected parameters, including the first coupling interval triggering Tdp, QRS duration of VE, VF, radiofrequency catheter ablation (RFCA) monotherapy or combination therapy, and ICD monotherapy/combination therapy (*P* < 0.05). No significant differences were observed between the two groups in terms of age, male sex, no structural heart disease, family history of sudden death, emotion, electrical storm, medication treatment alone, and death (*P* > 0.05). The scTdp originating from the Purkinje fibers is more likely to be generated in VF. RFCA and ICD monotherapy/combination therapy are more effective for scTdp originating from the RVOT and Purkinje fibers, respectively.

**Table 3:** Clinical characteristics of the different ventricular extrasystole's origins.

**Variable**	**RVOT (*n* = 25)**	**Purkinje fibers (*n* = 69)**	***P*-value**
Age (y, mean ± SD)	38 ± 11	38 ± 12	0.855
Male (*n*, %)	9 (43)	24 (52)	0.479
No structural heart disease (*n*, %)	25 (100)	68 (99)	1
Family history of cardiac disease or sudden death (*n*, %)	1 (5)	7 (15)	0.413
Emotion and exercise (*n*, %)	1 (4)	4 (6)	0.754
First coupling interval (ms, mean ± SD)	380 ± 70	274 ± 28	<0.001
QRS duration of ventricular extrasystole (ms, mean ± SD)	147 ± 8	131 ± 17	<0.001
VF (*n*, %)	12 (48)	54 (78)	0.005
Electrical storm (*n*, %)	0 (0)	6 (9)	0.295
RFCA Monotherapy/Combination therapy (*n*, %)	21 (84)	36 (52)	0.005
ICD Monotherapy/Combination therapy (*n*, %)	3 (12)	55 (80)	<0.001
Only medicines treatment (*n*, %)	2 (8)	12 (17)	0.422
Death (*n*, %)	1 (4)	5 (7)	0.888

RVOT, Right ventricular outflow tract; SD, Standard deviation; VF, Ventricular fibrillation; RFCA, Radiofrequency catheter ablation; ICD, Implantable cardioverter defibrillator.

**Figure 3:**
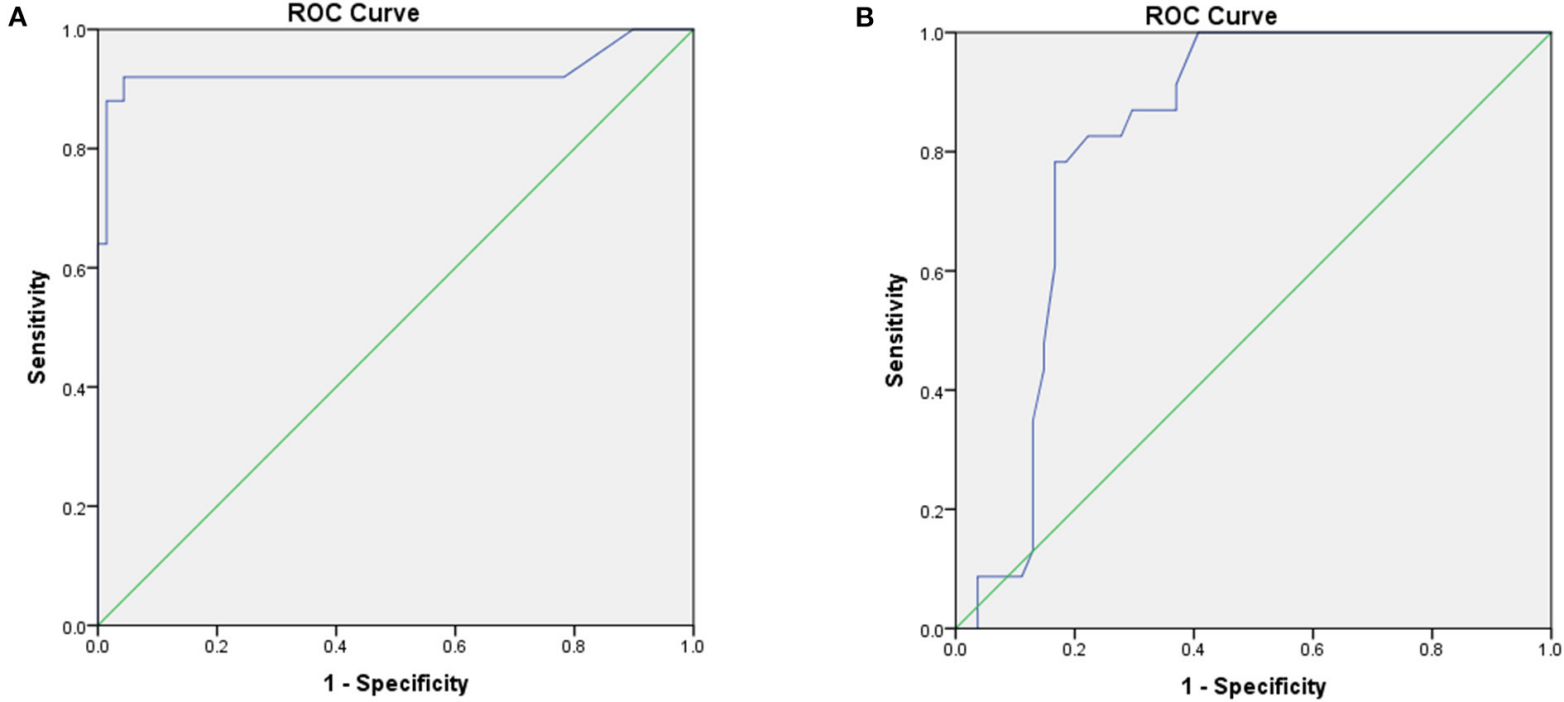
ROC analyses of the optimal cutoff values of the short-coupled interval triggering torsade de pointes (TdP) **(A)** and QRS duration of ventricular extrasystole (VE) **(B)** for predicting the right ventricular outflow tract (RVOT) origin.

ROCs were performed, and the optimal threshold was obtained when the Youden index was maximal. The optimal cut-off values of the first coupling interval triggering Tdp and QRS duration of VE for predicting RVOT origin were 319 ms (sensitivity 92%, specificity 95.7%) and 141 ms (sensitivity 82.6%, specificity 77.8%), respectively. ROC curves were established to assess the potential value of the first coupling interval triggering TdP and QRS duration of VE as electrocardiographic markers for predicting RVOT origin (Figure 3). There were remarkable differences between the first coupling interval triggering TdP and the QRS duration of VE, with an AUC of 0.928 [*P* < 0.001, 95% confidence interval (CI): 0.838–1.000], and 0.824 (*P* < 0.001, 95% CI: 0.731–0.917), respectively. The potential electrocardiographic markers were distinguished between different scTdp origin.

### Management and outcomes

**Figure 4:**
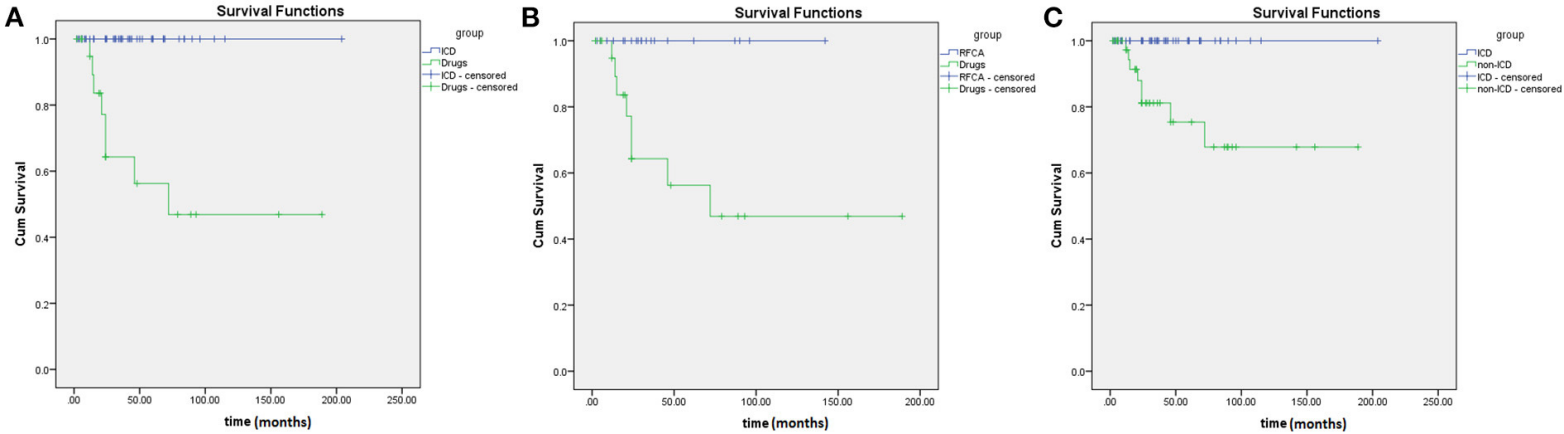
Comparison of Kaplan-Meier survival curves in short-coupled variant of torsade de pointes (scTdP) patients with different treatments. **(A)** implantable cardioverter defibrillator (ICD) vs. medication (*P* < 0.001); **(B)** radiofrequency catheter ablation (RFCA) vs. medication (*P* = 0.006); **(C)** ICD vs. non-ICD (*P* = 0.001).

**Figure 5:**
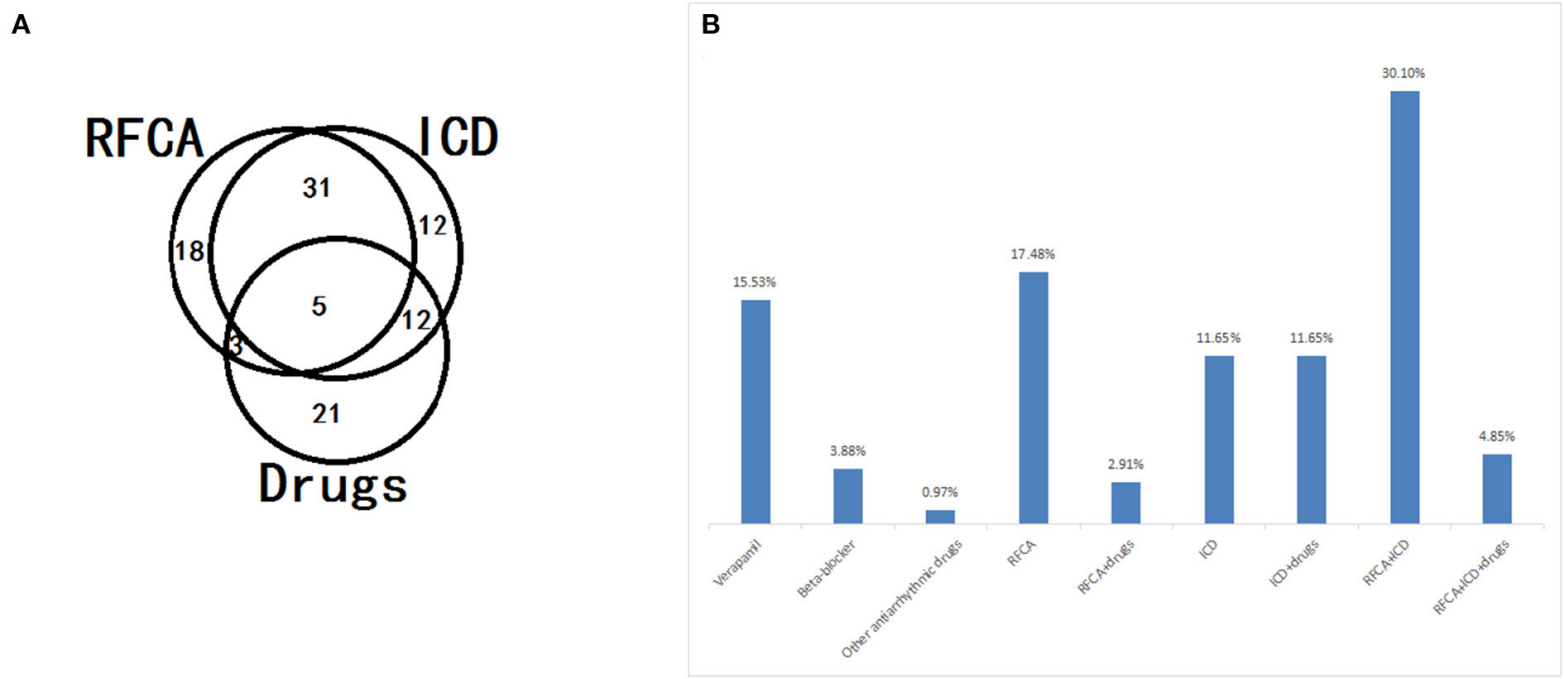
Application distribution **(A)** and constitution **(B)** of different treatment strategies, including radiofrequency catheter ablation (RFCA), implantable cardioverter defibrillator (ICD), and drugs, in patients with short-coupled variant of torsade de pointes (scTdP).

During the follow-up period, 58% of the selected patients underwent ICD implantation, 22% had arrhythmia recurrence, and 92% were alive. In this research, the median survival time was 72 ± 38 months in patients without ICD implantation, and the median survival time was 24 ± 5.5 months in patients with only medication. In the Kaplan–Meier curve (Figure 4), patients on medication showed higher mortality rates than patients with RFCA and ICD implantation (log-rank = 7.682, *P* = 0.006; log-rank = 19.7, *P* < 0.001). Moreover, patients without ICD implantation had higher mortality rates than those with ICD implantation (log-rank = 10.127, *P* = 0.001). It can be seen that both RFCA and ICD implantation may prevent the occurrence of sudden death, but the efficacy of ICD implantation is better (Figures 4, 5).

## Discussion

This systematic review analyzed the case reports of scTdp. Of the 36 studies searched in the databases, only 22 were selected and analyzed. The results of the quality assessment showed that all of the selected studies were sufficient. To the best of our knowledge, only a limited number of similar cases with sufficient information to recognize the major features of scTdP have been published.

Young patients (<60 years), without differences in sex, often had no high-risk factors for coronary artery disease, no structural heart disease, no family history of sudden death, and no emotional stress. A meticulous inspection of the 12-lead ECG should be performed to exclude any pathological ECG findings, including early repolarization J-wave syndrome phenotypes and QT syndromes (25). The ECG pattern was reportedly uniform, with a normal QT interval. The most valuable finding in the ECG was that a short coupling interval triggered TdP. However, there is no consensus in the literature regarding the normal value of the PVC coupling interval (6), which refers to the interval from the onset of normal QRS complex to the beginning of PVC on an ECG signal. Such ECG data should be interpreted with caution before claiming that TdP has a limited specificity.

The origin of the scTdP remains unknown. PVCs often precede VF but do not induce spontaneous VF, and malignant PVCs that induce VF usually originate in the same way (26). This indicates that the VF trigger mechanism has unique characteristics. The two primary sources of malignant PVCs triggering Tdp or VF are: (1) the Purkinje system and its distal arborized fibers and (2) the myocardium of the RVOT and left ventricular outflow tracts (LVOT) (27). In general, malignant PVCs originating from the Purkinje system are differentiated from their myocardial analogs by their coupling intervals. In this systematic review, we found that short PVC coupling intervals (<400 ms) indicate high-risk PVCs that trigger fatal arrhythmias. Among them, the malignant PVC coupling interval values of RVOT sources are usually ≥319 ms, and the short PVC coupling interval values of Purkinje sources are <319 ms. The cut-off value of a typically short QT interval is ≤ 320 ms, which is the main electrocardiographic marker of short QT syndrome (28, 29). The similar thresholds of the two different ECG entities can further confirm that Purkinje fibers as arrhythmogenic substrates play an important role in the occurrence of malignant ventricular arrhythmias. Moreover, the malignant PVC coupling intervals of left ventricular Purkinje sources are even shorter, usually ≤ 300 ms. It was demonstrated that the smaller the coupling interval of these extrasystoles, the greater the risk of spontaneous PMVT, and, therefore, of sudden death due to VF (6). Thus, the available data suggest that a shorter coupling interval of initiating PVCs correlates with the more malignant form of RVOT ventricular tachycardia (VT). However, a cutoff value that would reliably differentiate malignant RVOT VT from benign RVOT VT remains to be defined (30). Unlike the relatively short PVC coupling intervals associated with malignant arrhythmogenic PVCs, the absence of risk is not necessarily guaranteed by relatively long PVC coupling intervals. In general, malignant RVOT/LVOT PVCs have longer initiating coupling intervals than malignant Purkinje PVCs. Patients with malignant Purkinje PVCs more frequently present with VF than those with PVCs that originate from the RVOT. PVC morphology can also be pleomorphic.

The particular morphology of malignant PVCs [LBBB, left axis deviation, and late precordial transition (>V4)] suggests a Purkinje origin, one originating from the moderator band of the right ventricle. Notably, the coupling interval of the PVC that triggered VF was usually (however, not always) <300 ms. Malignant PVCs originating from the left ventricular Purkinje system localized along the ventricular septum and that morphologically resembled fascicular beats, presented with a relatively narrow QRS complex, an RBBB configuration, and a superior, inferior, or intermediate axis. In addition, the myocardium can give rise to PVCs that can produce a malignant phenotype. These sites correspond precisely to the regions of the myocardium that generate benign PVCs, for example, the RVOT and LVOT, and mirror the frequency of origin of benign PVCs. Malignant RVOT PVCs presenting with a relatively wide QRS complex, an LBBB configuration, and a right axis deviation, are more common than those that originate from LVOT sites. Our study also revealed that the LVOT PVC coupling interval triggering life-threatening arrhythmia is scarce (Figure 2).

A PVC-QRS duration of ≥153 ms and non-outflow tract origin (possibly related to a greater degree of dyssynchrony) were associated with the greatest risk of developing left ventricular dysfunction. In contrast, a PVC-QRS duration <153 ms and right ventricular outflow tract origin might be almost irrelevant to progressive left ventricular dysfunction, which is reversible and functional. Patients can benefit from ablation especially in ROVT PVC-induced cardiomyopathy, which is related to the amount and duration of PVC. In addition, our research confirmed that PVCs originating from the RVOT could be identified by the cut-off value of PVC-QRS duration (>140 ms) for predicting the triggering of PMVT or Tdp. Nevertheless, PVCs originating from the Purkinje fibers (<140 ms) could be distinguished from those originating from the RVOT. Almahameed et al. revealed that a PVC-QRS duration <140 ms was a significant feature of malignant PVCs in patients with unexplained syncope and apparently normal hearts (25). Therefore, the anatomical origin of the PVC and PVC-QRS duration is essential to predict left ventricular dysfunction and impending malignant arrhythmia. More importantly, current imaging modalities do not consistently and reliably differentiate between patients with PVC-induced cardiomyopathy and those with frequent PVCs and pre-existing non-ischemic cardiomyopathy.

TdP/VF in the absence of identifiable structural heart disease is usually the result of short coupled PVCs arising from the outflow tracts or the Purkinje system within either the right or left ventricles or, less commonly, from the ventricular myocardium. The typical PVCs initiating TdP/VF usually have a consistent QRS morphology and a short coupling interval and can be targeted for ablation to control the arrhythmia. For PVCs from the Purkinje system, the ablation target is a high-frequency Purkinje potential preceding the PVCs. When episodes are induced by short-coupled PVCs arising from the outflow tracts, the ablation target is the site of earliest ventricular activation (31). However, the detailed mechanism of scTdP remains unclear. Several reports have described triggered activity, abnormal automaticity, or reentry, as possible underlying mechanisms of idiopathic PMVT/VF originating from the Purkinje system (23). One study suggested that the scTdP mechanism might be reentry into the papillary muscles and the Purkinje network (15). Although the mechanism of benign idiopathic monomorphic VT arising from RVOT is considered to be triggered activity, that of idiopathic PMVT or TdP originating from RVOT is unknown, due to limited investigation of the electrophysiological characteristics during the ablation procedure. It is speculated that functional block and/or delayed conduction by rapid firing (caused by triggered activity or microreentry arising from a single focus) leads to chaotic ventricular conduction, thus causing PMVT and/or VF (8). However, it is also speculated that rapid firing from close multiple foci one after another produces polymorphic morphological changes in the QRS configuration, since other PVCs with slightly different QRS morphologies often appear after eliminating the initial target PVCs by RFCA (10, 30). In addition, scTdP is observed in the context of a particular autonomic nervous system profile, with low heart rate variability and a high sympathetic to parasympathetic ratio (9, 14).

A clinical hypothesis is that scTdP arises from the same genetic mutation with varying degrees of gene penetration, and consequently, different clinical expressions. Therefore, individuals with a more severe form of the disease could potentially develop malignant arrhythmias without necessarily having a coupling interval <300 ms (6). VE patients with short coupling may carry an uncommon syndrome, probably of genetic etiology, which can result in TdP. Most idiopathic PMVT/VF cases are sporadic; however, a subset of patients have a family history of SCD, which is suggestive of a genetic origin. Genetic screening of known genes responsible for arrhythmias has led to the identification of only a few ryanodine receptor 2 variants in a small percentage of cases (17). This suggests that these patients are genetically heterogeneous and that idiopathic PMVT/VF is possibly oligogenic in the origin of the Purkinje fibers, which could explain the low penetrance in families. Multiple genetic variants may be responsible, as is the case in other channelopathies (7). In addition, the scTdP may also be caused by many kind of inheretory channelopathies without structural heart abnormalities. Consequently, the further evaluation for genetic arrhythmia syndromes is recommended.

The diagnosis of scTdP is clinically and therapeutically important. It is critical to enhance the fundamental understanding of the relative importance of the PVC site of origin and PVC coupling interval in the triggering of fatal arrhythmias, as well as the dynamic interplay between the PVC and the underlying myocardial substrate. Several drugs, such as verapamil and β-blockers, and catheter ablation can reduce or suppress arrhythmic episodes in the short-term. However, this beneficial effect of medication does not prevent sudden death due to spontaneous and unpredictable arrhythmias (12). Decreasing the incidence of VF with localized ablation may reduce the requirement of defibrillation and ICD replacement, and improve the patient's quality of life (10). However, RFCA may be ineffective, because the same or similar PVCs may recur, or an ill-defined underlying electrical substrate or unidentified channelopathy may coexist. For example, idiopathic RVOT VT, a significant sign of arrhythmogenic right ventricular cardiomyopathy (ARVC), developed in one patient 10 years after RFCA. This suggests that RFCA seems to be effective in curing the malignant form of idiopathic VT arising from RVOT; however, a backup for ICD implantation is required in patients with the malignant form of idiopathic RVOT VT, especially in those with ARVC. In addition, when the PVCs can be identified, ablation is highly successful, but late recurrences are observed in ~10% of patients such that implantation of an ICD is prudent even if ablation is acutely successful (31). Moreover, the efficacy of catheter ablation has not been verified due to the lack of long-term follow-up data in the prevention of sudden death (30). Therefore, we strongly recommend the use of ICD therapy. However, limited data suggests that the subcutaneous ICD may not be a good therapy for these patients due to the higher risk of T-wave oversensing seenin this population (31). Due to the limited data in the literature on asymptomatic individuals, we chose to institute a clinical follow-up and prophylactic and empirical prescription of verapamil (6).

## Limitations

First, the electrocardiographic details of this rare heart rhythm disorder are undefined, and the underlying mechanism is unknown in sick individuals. Moreover, these reports were small-scale studies based on early restricted understanding, rather than large-scale clinical trials; hence, these data may not be decisive and relevant for the entire population. Second, there was a selection bias, as PVCs originating from the LVOT were not included. Third, the limitations of our results are due to incomplete information on some case descriptions. Further long-term follow-up studies are necessary to verify whether RFCA can prevent SCD.

## Conclusions

This systematic review was performed to synthesize and analyze case reports of scTdP. The main clinical features of scTdp include a normal cardiac structure and unexplained syncope in young patients. Short PVC coupling (<400 ms, especially ≤ 320 ms) and long PVC-QRS durations (<140 ms) are more likely to predict impending scTdp. Moreover, according to the cut-off values, we could distinguish between different origins of PVC triggering TdP and take effective treatments. Thus far, ICD implantation has been the only effective way to prevent SCD in these patients. Further large-scale and long-term follow-up studies, especially addressing the definitive diagnosis, risk stratification, and management of scTdP, are warranted.

## Data availability statement

The original contributions presented in the study are included in the article/supplementary material, further inquiries can be directed to the corresponding author/s.

## Author contributions

The GW was involved in the conceptualization. GW, HC, and CW performed data curation. GW, LZ, and HC performed formal analysis, performed the methodology, wrote, reviewed, and edited the manuscript. GW and LZ wrote the original draft. All authors have contributed to the manuscript and approved the submitted version.

## Funding

This study was supported by funding from the Medical and Health Development Program of Shandong Province (Grant No. 202103010621).

## Conflict of interest

The authors declare that the research was conducted in the absence of any commercial or financial relationships that could be construed as a potential conflict of interest.

## Publisher's note

All claims expressed in this article are solely those of the authors and do not necessarily represent those of their affiliated organizations, or those of the publisher, the editors and the reviewers. Any product that may be evaluated in this article, or claim that may be made by its manufacturer, is not guaranteed or endorsed by the publisher.

## Abbreviations

AUC: area under the receiver operating characteristic curve
ARVC: arrhythmogenic right ventricular cardiomyopathy
CI: confidence interval
ECG: electrocardiogram
ICD: implantable cardioverter defibrillator
LBBB: left bundle branch block
LVOT: left ventricular outflow tracts
PMVT: polymorphic ventricular tachycardia
PRISMA: Preferred Reporting Items for Systematic Reviews and Meta-analyses
PVC: premature ventricular complex
RFCA: radiofrequency catheter ablation
ROC: receiver operating characteristic
RBBB: right bundle branch block
RVOT: right ventricular outflow tract
scTdp: short-coupled variant of torsade de pointes
SCD: sudden cardiac death
VE: ventricular extrasystole
VF: ventricular fibrillation
VT: ventricular tachycardia.
